# Hydrogel-Enabled Delivery Systems for Agricultural Resilience: Controlled Release, Soil Interactions and Performance Constraints: A Review

**DOI:** 10.3390/gels12090785

**Published:** 2026-09-01

**Authors:** Cristofer Chambi, Julio Alegre Orihuela, María Pachés, Patricia Pacheco Umpire, Javier Montalvo Andia

**Affiliations:** 1 Programa de Doctorado en Ingeniería y Ciencias Ambientales, Universidad Nacional Agraria La Molina, Lima 15024, Peru; 2 Departamento de Suelos, Facultad de Agronomía, Universidad Agraria Nacional Agraria La Molina, Av. La Molinas/n, La Molina, Lima 15024, Peru; jalegre@lamolina.edu.pe; 3 CALAGUA—Unidad Mixta UV-UPV, Institut Universitari d’Investigacio’ d’Enginyeria de l’Aigua i Medi Ambient—IIAMA, Universitat Politècnica de València, Camí deVera s/n, 46022 Valencia, Spain; mapacgi@upvnet.upv.es; 4 Escuela Profesional de Ingeniera Ambiental, Universidad Católica San Pablo, Arequipa 04001, Peru; pbpacheco@ucsp.edu.pe (P.P.U.); jpmontalvo@ucsp.edu.pe (J.M.A.)

**Keywords:** water stress, biodegradable, bioactive microorganisms, encapsulation, hydrogel

## Abstract

Hydrogels (HGs) have emerged as promising multifunctional materials for sustainable agriculture due to their high water retention capacity and their ability to act as controlled-release platforms for agrochemicals, nutrients, microorganisms, and bioactive compounds. Their application has gained increasing attention in response to global challenges associated with climate change, water scarcity, soil salinization, and the low efficiency of conventional fertilizers, which contribute to environmental degradation and reduce crop productivity. This review provides a critical overview of hydrogel-based systems for agricultural applications, with particular emphasis on the encapsulation of bioactive components for soil remediation and crop protection under abiotic stress conditions. A systematic literature review following PRISMA guidelines was conducted using the Scopus database, resulting in the analysis of 548 studies published between 2003 and 2024. Bibliometric analysis revealed a marked increase in research activity since 2021, mainly driven by advances in water-retention technologies, nanocomposite hydrogels, controlled-release systems, and bioactive encapsulation strategies. The review discusses the main factors governing hydrogel functionality, including swelling behavior, crosslinking density, biodegradability, and interactions with soil–plant systems. Particular attention is given to recent developments involving the incorporation of microorganisms, nanoparticles, micronutrients, and agrochemicals into biodegradable hydrogel matrices to improve nutrient availability, mitigate salinity stress, and reduce agrochemical losses. Finally, the challenges associated with scalability, environmental stability, and field validation are discussed, highlighting the potential of hydrogel-based bioactive delivery systems for developing resilient and resource-efficient agricultural systems.

## 1. Introduction

According to the Food and Agriculture Organization of the United Nations (FAO), the global population is estimated to reach 10 billion by 2050 [1,2]. This accelerated demographic growth demands a significant increase in agricultural production, which has led to greater dependence on mineral fertilizers. However, it is estimated that only 50% of these fertilizers are effectively absorbed by plants; the remainder is lost through processes such as volatilization, soil-derived chemical reactions, and leaching into groundwater bodies [3,4]. This reveals that the current agricultural model is inefficient, unsustainable, and harmful to the environment. At the same time, the effects of climate change exacerbate this situation. According to the Fifth Assessment Report of the Intergovernmental Panel on Climate Change [5], global warming is associated with extreme events, sea level rise, alterations in crop growth and yield, and increasing pressure on water resources. Likewise, an intensification of processes such as desertification, soil degradation, and salinization has been documented, closely linked to the interactions between climate and land use [6]. According to the Global Map of Salt-Affected Soils (GSASmap) developed by the FAO [7], salinization affects 833 million hectares of subsoil (depths between 30 and 100 cm) and more than 424 million hectares of topsoil (0 to 30 cm) worldwide. More specifically, 397 million hectares are classified as saline soils and 434 million as sodic soils [8]. These areas are predominantly located in fragile ecosystems, with 37% found in arid deserts and 27% in dry steppes, both warm and cold [7].

An initial approach to soil salinization was reported by Gutierrez et al. [9], who analyzed soil degradation associated with agricultural lands in South American countries using maps describing soil erosion rates in 5 km grids for the years 1990, 2000, 2010, and 2020. Their results showed that intensive agriculture and the excessive use of chemical fertilizers constitute the main sources of soil degradation, manifested in processes such as salinization and soil contamination over recent decades, identifying two critical points with high salinity concentrations (>16 dS/m), one in the city of Arequipa in Peru and another in La Paz, Bolivia. Some of the effects of high salt concentrations include the induction of hyperosmotic shock and ionic imbalance, leading to nutritional disorders, oxidative stress, and even cell death [10]. Excess Na^+^ and Cl^−^ reduce the osmotic potential of the soil, hindering water uptake and causing dehydration and cytotoxic accumulation in plant tissues; moreover, they damage cells through the incorporation of salts into the transpiration stream [11]. Additionally, a decrease in germination and a significant reduction in agricultural yield have been reported, affecting variables such as number of pods and seeds, weight, height, biomass, and leaf area, all of which are correlated with soil salinity concentration [12,13].

To improve and address these challenges to crop productivity, it is crucial to develop innovative alternatives that promote more sustainable and resilient agriculture, focused on enhancing productivity without compromising natural resources [14,15]. Some techniques aimed at optimizing nutrient absorption efficiency in crops are based on polymeric materials applied in different fields, such as medicine [16], engineering, and agriculture [17,18].

One of the most promising applications of these polymeric materials in agriculture is the development of hydrogel (HG) spheres, also referred to as beads, made from polysaccharides that can be of biological or chemical origin. These materials have gained relevance in recent decades in the agricultural field because of their functional properties [19,20]. Although they present a morphology similar to conventional polymeric granules, these structures offer the advantage of encapsulating active ingredients and nanoparticles within them, allowing for a controlled release of bioactive compounds [21]. This slow-release capability reduces physicochemical soil degradation processes [15], agrochemical leaching, and abiotic stress due to salinity [22]. In addition, HG spheres can be used to encapsulate bioactive compounds and bacteria with bioprotective functions (bioremediation) under both biotic and abiotic stress conditions. In this way, contamination during storage and transport stages is reduced, facilitating plant growth [23].

In this context, the present review aims to provide a comprehensive overview of the encapsulation processes of bioactive compounds in HGs for their application in agriculture and the remediation of soils degraded by abiotic stress (salinity) and water stress to improve controlled release and nutrient availability efficiency in crops. First, a bibliometric analysis of HGs and their agricultural applications was conducted. Based on this meta-analysis, the role of HGs as agrochemical delivery systems is discussed, followed by the current state of this technology and an analysis of the research landscape and the level of maturity in the use of HGs in agriculture. Subsequently, the main factors affecting their functionality and recent trends in the encapsulation of microorganisms, bioactive compounds, and/or agrochemicals are identified. Finally, the challenges associated with the use of HGs in real agricultural environments are identified, and the opportunities they offer to increase productivity and mitigate the effects of salinity and water stress are explored. This review delves into the use of bioactive components as a strategy for agricultural intensification under stressful conditions. The novelty of this review lies in its focus on the integration of bioactive components encapsulated in HGs, offering a detailed analysis of how this combination can intensify sustainable agriculture and contribute to the remediation of soils affected by salinity and water scarcity.

## 2. Materials and Methods

### 2.1. Literature Search

On 15 June 2025, a systematic literature search was performed in the Scopus database. The review was conducted in accordance with the Preferred Reporting Items for Systematic Reviews and Meta-Analyses (PRISMA) guidelines [24]. The following keywords were used: “hydrogel synthesis” AND “agriculture” in the title, abstract, and keywords (Supplementary Materials, Table S1). Duplicate articles, review articles, conference proceedings, and articles written in a language other than English were excluded. Although this strategy ensured a consistent and reproducible dataset, the use of a single bibliographic database and predefined search string may have excluded relevant studies indexed in other databases or published using alternative terminology.

### 2.2. Selection Criteria

After the analysis of abstracts, articles that did not address the encapsulation of microorganisms or agrochemicals for their application in soil within matrices of biological or synthetic origin for agricultural purposes were excluded.

In addition, a patent search was conducted in the Espacenet database using the keywords “hydrogel synthesis” AND “agriculture” to complete the collected bibliography (Supplementary Materials, Table S1). The search was performed independently.

### 2.3. Data Analysis and Research Field

**Figure 1:**
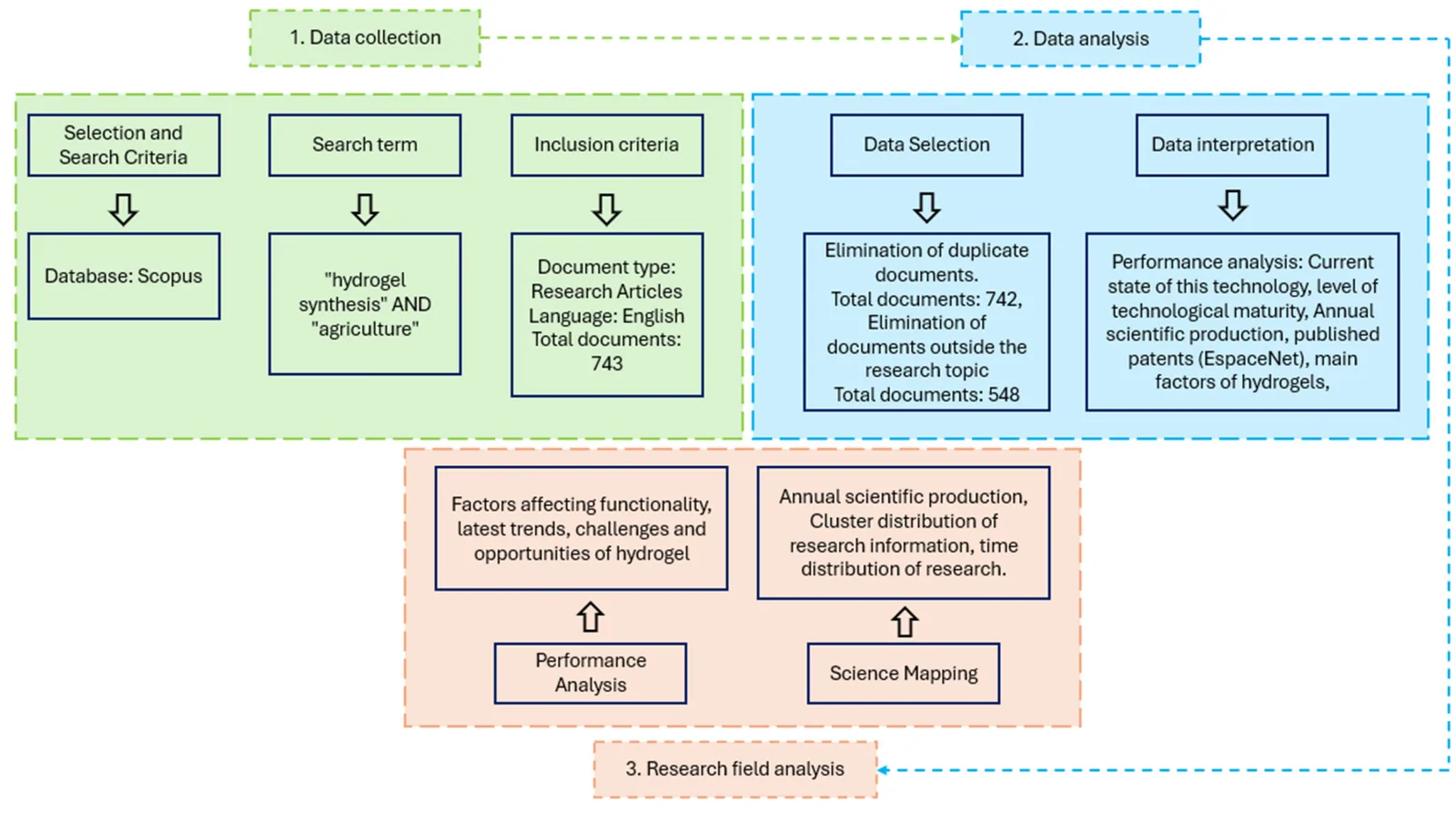
Diagram for the descriptive analysis based on the PRISMA methodology and VOSviewer software.

A search was conducted in the Scopus database, which is recognized as one of the reliable sources for data collection [25]. For the development of research on HGs in agriculture, the VOSviewer version 1.6.20 software was used, which allows the construction of bibliometric maps based on co-occurrence relationships. In particular, keyword co-occurrence maps were generated, organized into clusters, and defined as groups of terms closely related to each other according to the frequency with which they appear together in the documents. Likewise, a temporal visualization was developed to identify the evolution of these terms over time. The workflow of the exploratory and descriptive analyses is presented in Figure 1.

## 3. Results and Discussion

The search identified 743 records and only one duplicate article, resulting in a final total of 742 articles. A preliminary reading of the abstracts was conducted to filter those that did not meet the selection criteria, eliminating articles not related to agricultural applications or focusing on pharmacology and medical studies. After applying the selection criteria, 548 articles remained, which will be used to develop the review article. Following this filtering process, a comprehensive overview of HGs in agriculture is presented, structured as follows: (1) thematic structure and evolution of research on HGs in agriculture; (2) role of HGs as delivery systems; (3) research landscape and level of technological maturity; (4) factors affecting the functionality of hydrogels in agriculture; (5) recent trends in the encapsulation of microorganisms, bioactive compounds, and agrochemicals; and (6) challenges faced by hydrogels in agriculture.

### 3.1. Thematic Structure and Evolution of Research on Hydrogels in Agriculture

**Figure 2:**
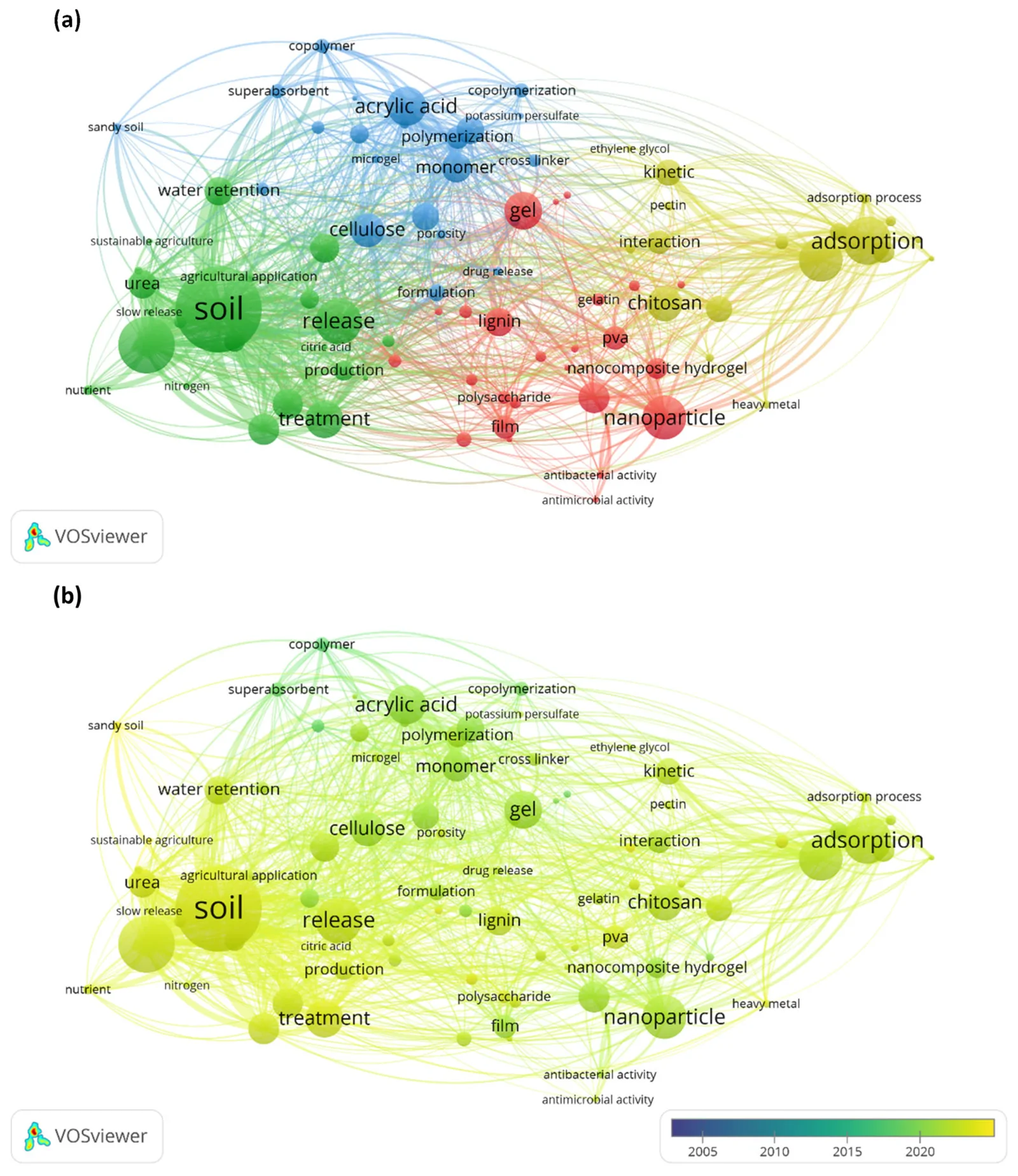
Network graph of HGs in agriculture from 2003 to 2024, using VOSviewer. (**a**) Distribution by information clusters of research on HG trends in agriculture; (**b**) temporal distribution of research information on HGs in agriculture from 2003 to 2024.

The VOSviewer software is an innovative tool for mapping bibliographic information and graphical representation according to keyword similarities. In this study, bibliometric analysis enabled the identification of the main research trends in the field of HGs applied to agriculture during the period 2003–2024. The results were grouped into four main clusters (Figure 2a), which represent the information available in the bibliographic repository over the last twenty-one years. The first cluster (green) encompasses the literature related to the application of HGs in soil, such as water retention and release rates. The second cluster (red) focuses on nanoparticles (from the synthesis of nanogels with materials such as lignin and other polysaccharides to nanocomposite hydrogels). The third cluster (yellow) is related to adsorption processes in soil (kinetics, interaction, and adsorption of heavy metals). Finally, the fourth cluster (blue) refers to the main formulation components of HGs (monomer, formulation, copolymerization, and crosslinking).

In addition, a trend analysis over time was applied (Figure 2b) to define recent research areas. The analysis revealed that the first cluster (approaches related to soil application, treatment of sandy soils for water retention, and the slow release of some agrochemicals) corresponds to the most recent studies (yellow ≥ 2020), whereas studies conducted between 2015 and 2020 were more focused on HG formulation. Finally, current trends are more related to application technologies in agriculture, such as superabsorbents and copolymerization.

### 3.2. The Role of Hydrogel Technology as a Delivery System in the Development of Agriculture

**Figure 3:**
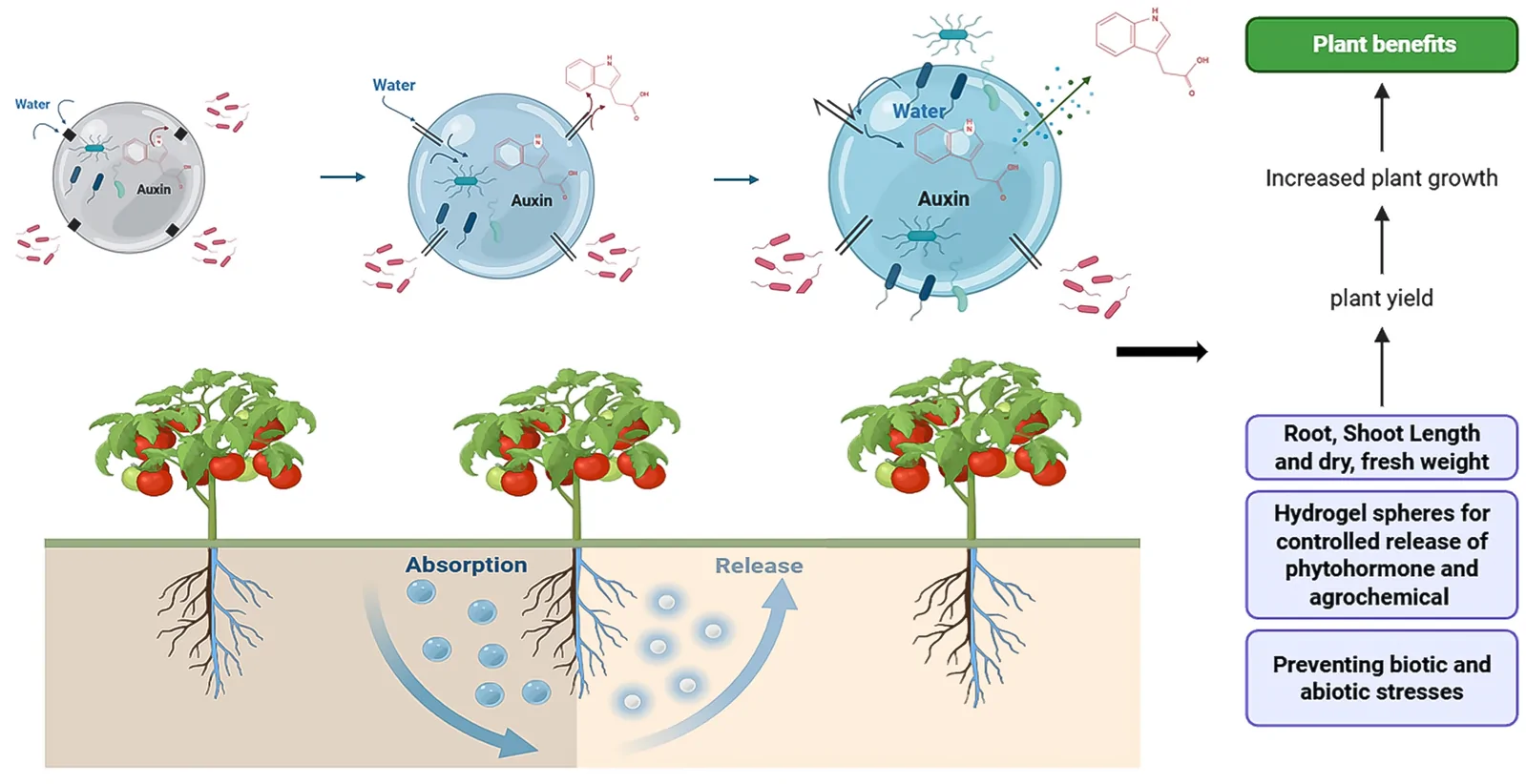
Schematic models of HGs applied in agriculture for the controlled release of nutrients, pesticides, and plant growth-promoting bacteria.

Hydrogels are three-dimensional polymeric materials with a high water absorption capacity. Among them, superabsorbent hydrogels are a specific class of hydrophilic polymer networks capable of absorbing and retaining large amounts of water or aqueous solutions while maintaining their structural integrity without dissolving [26,27]. These superabsorbent structures can retain large volumes of water and gradually release the retained content, allowing water and bioactive compounds to be available in a sustained manner in the root zone of plants, which is essential for plant development under abiotic stress conditions [28], as schematically shown in Figure 3.

In conventional irrigation systems, a significant portion of the applied water is lost through evaporation or percolation into deeper soil layers, which limits its availability to roots and contributes to crop water stress [29,30]. In this scenario, the use of HGs as controlled delivery systems represents an efficient alternative to conserve water in the soil and reduce irrigation frequency while facilitating a gradual and localized delivery of nutrients, phytohormones, pesticides, or beneficial microorganisms [31].

The functionality of HGs in agricultural applications goes beyond their water retention capacity. It is possible to develop formulations with controlled-release properties that encapsulate active ingredients and deliver them in a regulated manner, thereby optimizing their effectiveness and reducing the environmental impacts caused by the excessive use of pesticides [32]. A common approach is the use of polymeric matrices in which active compounds are dissolved or homogeneously distributed, allowing sustained release as the HG swells or degrades.

By reducing the loss of agricultural inputs through leaching or evaporation, HGs contribute to cleaner, more cost-effective, and climate-resilient agriculture [15]. Their incorporation into intensive production systems can play a key role in the transition toward sustainable agricultural models by improving water use efficiency, reducing agrochemical pollution, and increasing productivity in degraded soils or those affected by water stress.

### 3.3. Hydrogels in Agriculture: Research Landscape and Level of Technological Maturity

**Figure 4:**
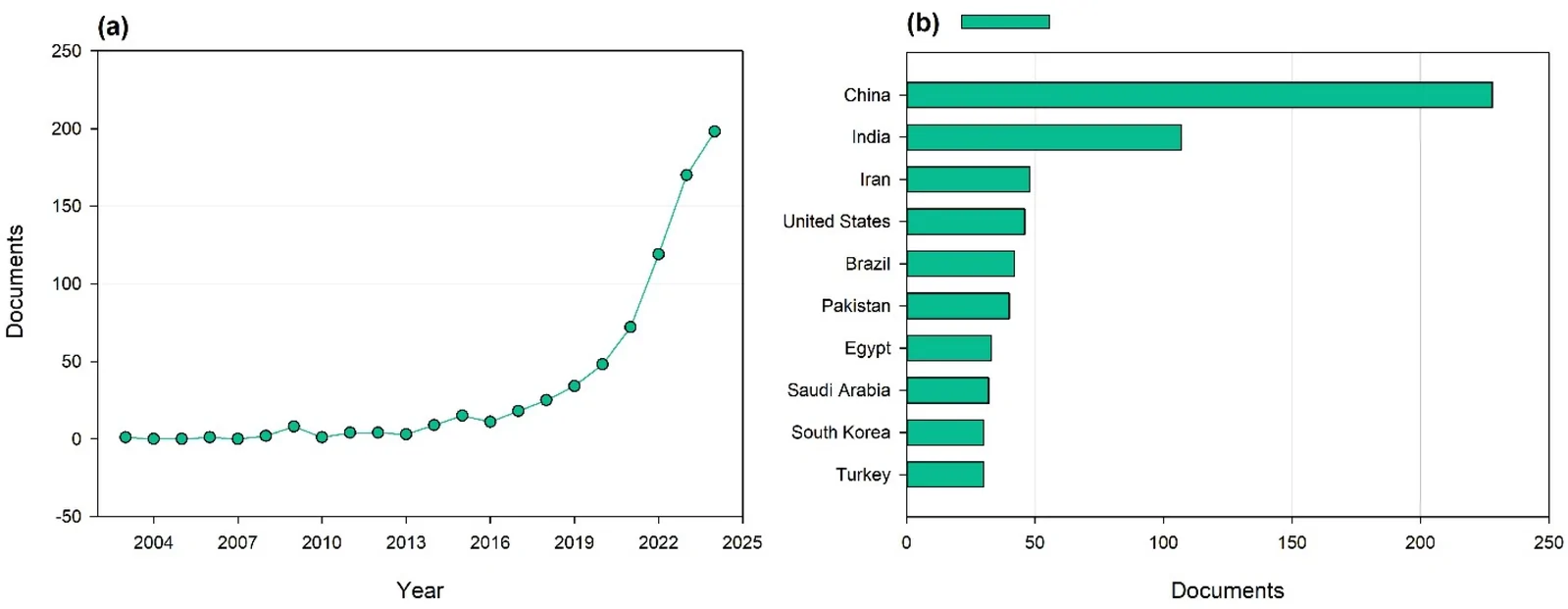
(**a**) Number of documents published on “hydrogel synthesis” AND “agriculture” per year and (**b**) number of documents published on “hydrogel synthesis” AND “agriculture” by country or region. Source: Scopus database using the keyword “hydrogel synthesis” AND “agriculture”.

As shown in Figure 4a, the field of HG synthesis applied to agriculture has experienced notable growth in research activity over the last two decades, beginning around 2003. In its early years, scientific production in this area was limited, reflecting the emerging nature of this technology and its initial stage of exploration. However, since 2021, an exponential increase in the number of publications has been observed, with 559 of the 743 retrieved studies (75.2%) published between 2021 and 2024, demonstrating a growing interest in the use of HGs as a technological solution to mitigate water stress in crops. This interest was consolidated in 2024, a year in which approximately 198 publications were recorded, marking a significant peak in annual scientific production.

This accelerated growth is likely due to the global need to develop more sustainable and efficient agricultural solutions, highlighting the potential of HGs as superabsorbent materials. These systems not only improve water retention but also enable the encapsulation of bacteria, nanoparticles, micronutrients, and other bioactive compounds [33]. In addition, their synthesis from low-cost polymeric materials of both biological and chemical origins reinforces their applicability in diverse agricultural contexts.

During 2019–2023, the average number of annual publications was approximately 89, demonstrating consistent and sustained interest in this field. In terms of geographical contribution, China and India lead scientific production, with approximately 228 and 107 publications, corresponding to 30% and 14%, respectively, reflecting a significant investment in research on HGs and biotechnological encapsulation applied to agriculture (Figure 4b). The success of these countries in research on HG applications is linked/related to the Green Revolution in Asia, which drives investment in advanced agricultural technologies [34]. This group is followed by the United States with nearly 46 publications, while other relevant countries, such as Iran, Pakistan, Brazil, and South Korea, have generated more than 30 publications each. Other countries within the European Union have lower participation in publications specifically focused on HGs applied to agriculture, despite several European countries having a relevant role in HG research in broader fields (such as materials science or biomedicine) [35].

This geographical distribution highlights an international consensus on the potential of HGs as a promising pathway to reduce the water footprint, improve crop resilience, and contribute to global food security in large-scale agricultural systems. Although Latin America as a whole shows lower participation in specific publications on HGs applied to agriculture, Brazil has emerged as one of the countries in the region that has promoted this area of research, partly due to the structural importance of agriculture in its economy and the predominance of first-generation crops with a high demand for solutions for water use efficiency [26]. This fact opens the way for the development of biotechnological solutions adapted to its agroclimatic conditions and productive needs for agroexport [36].

The sustained growth of publications in the last decade highlights the increasing relevance of this technology, not only for its technical effectiveness but also for its low cost and compatibility with biodegradable materials, which allows the incorporation of microorganisms or bioactive particles that promote soil health. Despite the growing interest, most current studies on HG synthesis remain at low technology readiness levels (TRLs), mainly between TRL 3 and 4, corresponding to laboratory research and prototype validation [37,38,39]. Only a limited number of studies have reached TRL 6 [40,41,42], where functionality is demonstrated at pilot plant scale; however, these studies are still scarce and require more time to validate their performance under real conditions, optimize process scalability, and ensure the technical and economic feasibility of the developed systems. In general terms, research on HGs spans from TRL 1 to TRL 6, covering conceptual development to product verification in controlled environments. Progress toward higher levels (TRL 8–9), necessary for commercial implementation, involves overcoming challenges related to industrial scalability, operational stability, and profitability, as well as aligning business interests with sustainability criteria and compliance with environmental regulations. This technological gap highlights the need for greater investment in research, innovation, and public–private collaboration to facilitate the transition of HGs from the laboratory to large-scale industrial applications. Although the challenge is significant, it can be overcome with the coordinated support of governments, research institutions, and companies committed to the development of sustainable agricultural technologies.

It is worth noting that the search conducted in the Espacenet database, using the keyword “hydrogel synthesis” AND “agriculture”, reveals an active landscape in the field of patents, mainly focused on improving water absorption and on the encapsulation of bioactive particles through the development of superabsorbent materials with agricultural applications. A total of 58 patents were identified, of which some are presented in Table 1. These patents were classified into five categories according to the focus of HG development: (i) synthesis of HGs for agricultural applications and other areas (31.03%); (ii) encapsulation of nutrients and other functional compounds (13.79%); (iii) development of additives or design modifications aimed at improving HG performance (24.14%); (iv) synthesis of HGs used as adsorbents of metals and other contaminants (6.90%); and (v) other patents (24.14%).

In general terms, these inventions propose various innovative formulations that combine materials of biological origin, such as chitosan, starch, and functional compounds, including graphene derivatives, with the aim of synthesizing HGs with a higher crosslinking capacity, which improves their performance as superabsorbent agents. Other studies indicate that these HGs incorporate bacterial genetic resources, microorganisms, or other biological sources, allowing them to confer additional bioactive properties and expand their functional potential in agricultural applications [43].

Many of these patents detail specific technical procedures for the optimization of parameters such as temperature, component ratios, and gelation conditions, as well as their application not only in agriculture but also in sectors such as forestry. In addition, some applications address the capacity of these HGs to store salts, fertilizers, and agrochemicals, functioning as controlled-release systems. However, in several of these cases, the functional properties mentioned are not experimentally validated and remain at a theoretical or conceptual stage. This trend in the patent field reflects the continuous interest in turning HGs into functional, low-cost, and adaptable materials capable of improving crop conditions under water stress and other abiotic factors, in line with the global push toward the development of green, efficient, and bio-based technologies.

### 3.4. Classification of Hydrogels

The classification of HGs is not unidimensional; rather, it encompasses multiple categorization schemes, ranging from the nature of the synthesis raw materials, polymer composition, crosslinking process, structural architecture, morphology, and electrical charge of the polymeric networks to their responsiveness to environmental stimuli and the specific requirements of each application area.

Based on polymeric origin, hydrogels are categorized into natural, synthetic, and semisynthetic or hybrid systems [44]. Natural hydrogels are composed of biological macromolecules derived from nature, such as alginate, chitosan, cellulose, collagen, gelatin, and pectin, among others [45,46]. On the other hand, synthetic hydrogels are produced from petroleum-derived monomers; among the most widely used in agriculture are polyacrylamide (PAM), poly(acrylic acid) (PAA), poly(vinyl alcohol) (PVA), poly(ethylene glycol) (PEG), 2-hydroxyethyl methacrylate (HEMA), and poly(N-isopropylacrylamide) (PNIPAM) [47], which stand out for their extraordinary superabsorbent swelling capacity.

Hybrid or semisynthetic systems arise from the chemical modification of natural polymers, such as the carboxymethylation of cellulose to graft synthetic chains onto a biodegradable backbone [48]. This category also encompasses the incorporation of inorganic fillers (such as clays, biochar, and nanosilica, among others) into the polymeric matrix [49]. Regarding their macromolecular composition, these structures are subdivided into homopolymers (a single monomeric species), copolymers (two or more monomers), Interpenetrating Polymer Networks (“IPNs”, two entangled networks), and semi-Interpenetrating Polymer Networks (“semi-IPNs”, linear chains within an uncrosslinked network) [50].

Hydrogels are also distinguished by their internal physical structure (amorphous and semi-crystalline): amorphous chains are randomly oriented, whereas semi-crystalline systems exhibit ordered, crystalline domains embedded within an amorphous matrix (as in the case of nanocrystalline cellulose hydrogels) [51].

Categorization based on encapsulation morphology in agricultural soil applications represents a critical factor governing the release of bioactive compounds. Two main configurations are distinguished: matrix microspheres (entrapment matrices), which distribute the active ingredient homogeneously for continuous release via diffusion and erosion, and core–shell microcapsules [52]. The latter house the biomass within a core protected by a polymeric shell that acts as a physical shield against desiccation, osmotic stress, and UV radiation, thereby optimizing system permeability and microbial survival.

The coexistence of these distinct classification criteria is neither superficial nor merely descriptive; each physicochemical category imposes direct constraints on mechanical stability, swelling kinetics, biodegradation rate, osmotic response, and compatibility with the encapsulated active ingredient or biological organism. The interplay between hydrogel classification and soil performance demonstrates that selecting the hydrogel class is not an arbitrary parameter but rather a central design decision that dictates the success of a controlled-release system, particularly when the hydrogel serves as a carrier for living microorganisms.

### 3.5. Synthesis Methods

The synthesis of HGs can be carried out through a wide variety of methodologies that are generally classified as physical and chemical techniques. Physical techniques include freeze–thaw cycles [53,54], ionic interactions [54], and aging [55]. These strategies stand out for eliminating or minimizing the use of toxic reagents, which is a key factor for material sustainability.

On the other hand, chemical techniques comprise various polymerization methods (bulk, radiation, and solution copolymerization) [56], grafting [57], and click chemistry [58], which offer superior scalability indicators. Recently, innovative technologies have emerged, such as emulsion templating, enzymatic crosslinking, and 3D printing, enabling the synthesis of HGs with high-precision structural control [59,60]. Ultimately, selecting the ideal synthesis method depends largely on the required type of crosslinking, the chemical nature of the polymer, the intended final application, and the desired biocompatibility.

### 3.6. Factors Affecting the Functionality of Hydrogels in Agriculture

**Figure 5:**
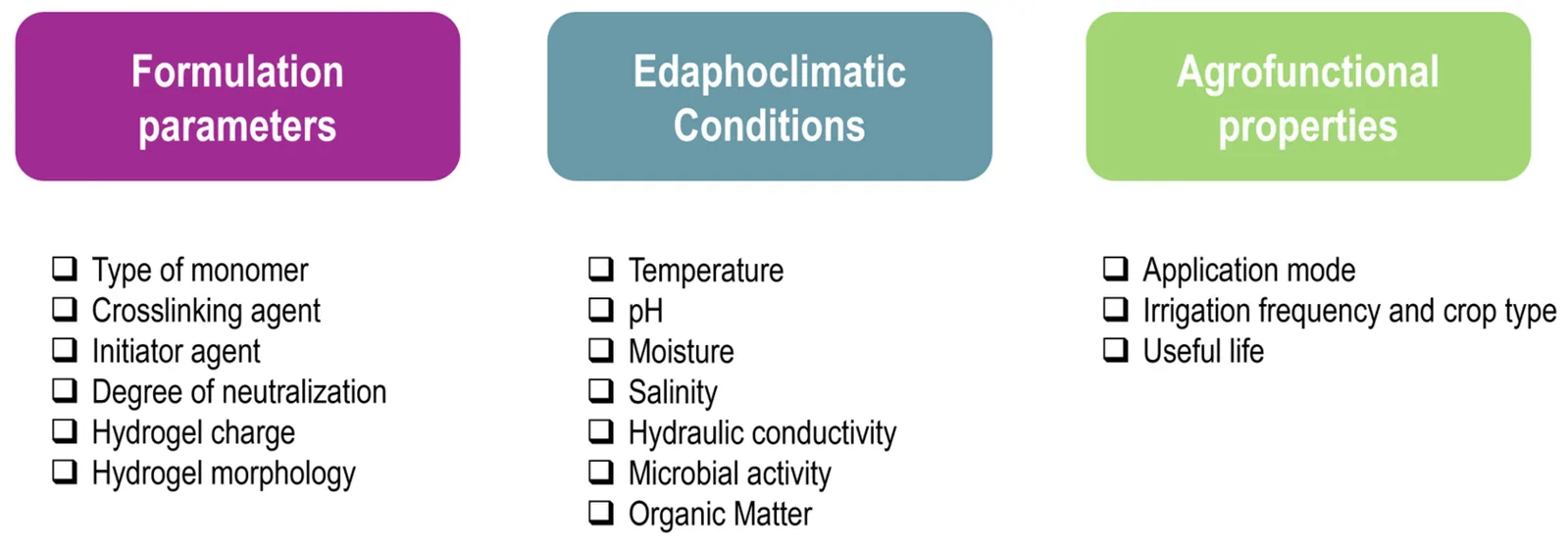
Comprehensive summary of the variables that require careful optimization to improve the efficiency of HG generation or production in sustainable agriculture.

The functionality of HGs in agricultural applications depends on multiple factors that determine their field performance. These factors include the formulation parameters, edaphoclimatic conditions, and agrofunctional properties. Figure 5 provides an integrated overview of the variables that require careful consideration and control to improve the functionality of HGs in the field.

#### 3.6.1. Key Formulation Parameters

The design of functional HGs depends on several formulation parameters that determine their physicochemical properties and performance in agricultural applications. Among the most relevant are the type of monomer, the polymerization process, and the release kinetics of the encapsulated compounds [26]. In particular, the type of monomer constitutes the structural basis of the HG, as it defines the nature and density of the hydrophilic groups present in the polymeric network [33]. This characteristic directly influences the water absorption capacity, such that a higher density of hydrophilic functional groups promotes an increase in water retention and material performance [28].

The second parameter is the crosslinking agent, which binds the monomer chains together through covalent bonds, generating a three-dimensional network structure. N,N’-Methylenebisacrylamide (MBA) plays a crucial role in the transformation of the polymer from a liquid state to a gel or solid [61]. In addition, these agents can modify key properties of the HG, such as elasticity, viscosity, solubility, strength, and toughness [62]. In addition to covalent crosslinking, ionic and physical crosslinking are widely employed, particularly in polysaccharide-based hydrogels. Ionic crosslinking, commonly used in alginate, chitosan, and carboxymethyl cellulose hydrogels, relies on multivalent cations to stabilize the polymeric network and regulate the mechanical strength, porosity, and swelling behavior [63,64]. Physical crosslinking is based on reversible non-covalent interactions, such as hydrogen bonding, electrostatic interactions, and chain entanglements, which provide self-healing capabilities [65,66]. For example, chitin nanofiber-based hydrogels have been physically crosslinked through the thermal decomposition of urea, which promotes the deprotonation of amino groups, reduces electrostatic repulsion, and induces nanofiber entanglement to form a stable three-dimensional network, demonstrating their potential as slow-release fertilizers for improving soil health [67].

The third key parameter in the formulation is the initiator agent, which is essential for the polymerization of monomers. This compound, such as benzoyl peroxide or ammonium persulfate, activates the formation of polymer chains [17]. For example, Liu et al. [68] used ammonium persulfate as an initiator for the synthesis of a bio-based superabsorbent HG that exhibited a water retention of 88.8% after 8 h.

The fourth parameter is the degree of neutralization (DN), which directly influences the structure and functionality of polymers by modifying the amount of ionic groups and their behavior in solutions. An adequate DN improves crosslinking and water absorption; however, an excess DN can generate negative effects, such as self-crosslinking or ionic shielding [69,70].

The fifth parameter is the HG charge. Based on this, they can be classified as ionic and non-ionic [71]. Ionic HGs interact electrostatically with cations (amines) or anions (carboxylates or sulfonates) present in the medium. For example, HGs based on acrylic acid or 2-acrylamido-2-methylpropane studied by Haripriya and Vijayakrishna et al. [72] present carboxylate groups that confer an anionic character and a high water absorption capacity, as well as contaminant adsorption systems. Another study conducted by Hydayat et al. [73] developed an alginate-based HG with acrylic acid to encapsulate struvite and improve the controlled release of nutrients in saline soils. On the other hand, non-ionic HGs do not possess a net charge, and water absorption occurs mainly through hydrogen bonding [74]. One approach is the study by Omar and Alsharaeh et al. [75] in which an HG based on polyacrylamide was evaluated to improve water retention in sandy soils, being a non-ionic HG. Other components, such as poly (vinyl alcohol), a non-ionic polymer, and acrylic acid, have demonstrated greater resistance to salinity and higher water absorption capacity [76,77]. Another example is sodium polyacrylate, an anionic polymer, used as an HG, which has been successfully used in agricultural trials in sandy soil. In this type of soil, the use of HGs not only increased plant growth but also facilitated nutrient absorption and accumulation, thus improving the fertilizer use efficiency [78].

The final parameter corresponds to HG morphology, including thickness, size, porosity, and surface charge, which directly influence its performance for water absorption and/or the release of bioactive compounds [40,79,80]. It has been demonstrated that well-defined porous structures favor both water retention and controlled release of compounds [81]. Considering these properties, sodium alginate stands out as one of the most widely used polysaccharides in the synthesis of biodegradable HGs because of its biocompatibility, although it presents limited mechanical strength [37]. In one approach, Abdukerim et al. [82] evaluated cucumber seed coating using an alginate-based HG loaded with *Bacillus subtilis* ZF71 for the control of root rot caused by *Fusarium*. The results showed the formation of a biofilm-like structure with a high density of viable cells, which improved system efficacy and reduced disease incidence by 53.26%.

#### 3.6.2. Influence of Edaphoclimatic Conditions on the Functionality of Hydrogels

The second essential factor is related to the edaphoclimatic conditions of the HG application site. These conditions play a crucial role in HG performance due to the variety of soil types and climatic conditions. For example, it is known that temperature strongly impacts the release of encapsulated nutrients, being directly proportional, where higher temperature leads to greater release [79]. Soil pH is considered a key variable in soil chemistry, as it determines the availability of nutrients for plants [83]. In HGs, their activity is also affected; for example, an acidic pH is suitable for the release and dissociation of different functional groups, whereas an alkaline pH reduces swelling and results in a lower rate of diffusion or nutrient release [20]. The pH-responsive swelling behavior of hydrogels is strongly governed by their chemical composition, particularly by the type and density of ionizable functional groups and the architecture of the polymeric network. In anionic hydrogels containing carboxylate or sulfonate groups, swelling generally increases as pH rises above the pKa due to the deprotonation of functional groups, which increases electrostatic repulsion and osmotic pressure within the network [84,85]. Conversely, cationic hydrogels rich in amino groups exhibit greater swelling under acidic conditions because protonation increases the positive charge density, whereas swelling decreases under alkaline conditions as the polymer chains contract [86]. Ampholytic hydrogels containing both anionic and cationic groups exhibit more complex swelling behavior because both ionization mechanisms occur simultaneously. In addition, network composition also plays an important role, since increasing crosslinking density generally reduces free volume and water uptake, whereas the incorporation of hydrophilic or ionizable monomers enhances pH sensitivity [87,88]. Under agricultural conditions, dissolved salts and multivalent cations may further reduce swelling by charge screening or by forming additional ionic crosslinks, thereby affecting the release of water, nutrients, and agrochemicals from the hydrogel matrix.

Soil moisture conditions at the time of HG application are also relevant. The application of HG under optimal moisture conditions improves germination rate and root growth [31]. In addition, soil parameters such as organic matter content, salinity, and soil type affect HG biodegradability. For example, when the organic matter content in agricultural soils is low, biodegradability is higher due to the use of HG as a carbon source by soil microorganisms [89]. In contrast, biodegradability is low in natural soils or in saline soils because the acidic pH and high salt concentration of these soils, respectively, can slow down HG biodegradation [90].

Finally, another relevant soil aspect is saturated hydraulic conductivity, a key parameter in agronomy and soil science. The effect of HG addition on this property has shown inconsistent results, as it largely depends on the interaction between soil texture and HG concentration. In some cases, its incorporation can lead to pore blockage and an increase in friction between soil particles, the HG, and water, which ultimately reduces water infiltration into the soil profile [91,92].

Soil microbial activity can be modified by the incorporation of HGs, particularly when they function as encapsulation matrices for microorganisms or bioactive compounds, thus influencing the structure and diversity of the microbial community. In the rhizosphere, HGs generate beneficial effects by promoting microbial interactions and associated functional processes. For example, Wang et al. [93] demonstrated that the hexavalent chromium content could be reduced by 17% by bacteria such as *B. subtilis*, while also significantly increasing the abundance of other functional bacterial populations associated with heavy metal stress tolerance (Proteobacteria, Actinobacteria, and Chloroflexi), which strongly promotes interactions and correlations among microorganisms. Similarly, Abdukerim et al. [82] evaluated an HG incorporated with *B. subtilis* ZF71 applied to cucumber seeds for the control of root rot caused by *Fusarium*, observing a reduction in disease incidence of 53.26%. Other effects derived from the use of HG include an increase in basal soil respiration and microbial activity in soil [94].

#### 3.6.3. Agrofunctional Factors in the Application of Hydrogels

The last determining factor of HG performance is related to agrofunctional properties. These refer to the mode of application, dosage, type of crop, and type of HG. For example, Kumar et al. [95] evaluated the productivity of *Zea mays* using a Pusa HG developed by the Indian Agricultural Research Institute (New Delhi) with a mixture of HG and dry soil in a 1:10 w/w ratio at a depth of 10 cm, with two different doses, 2.5 and 5 kg ha^−1^, and a control group without HG. The results of the growth and productive yield of *Z. mays* showed that at higher doses (5 kg ha^−1^), the yield was 10% higher. In another study conducted by Zangana & Aljburi et al. [96], the effect of HG (Real Fine) was evaluated before the sowing of *Triticum aestivum* L. under different soil water conditions. A total of 24 g of HG was applied per 3 m^2^ at an approximate depth of 7–8 cm, applied in the sowing lines, under sufficient irrigation conditions (sprinkler) and water stress conditions. The treatment with HG under sufficient irrigation conditions achieved a grain yield rate of approximately 3.3 t ha^−1^, compared to 2.8 t ha^−1^ without HG.

In regions characterized by low precipitation and sandy or sandy loam soils, limited water retention capacity and high water loss through percolation represent critical factors that restrict crop growth and productivity [97]. Under these conditions, the use of HGs has proven to be a strategy to improve water availability in the root zone and mitigate water stress. For example, Yang et al. [98] evaluated the combined effect of HGs applied to the soil and fulvic acid as a foliar treatment in *Zea mays* crops established in a semi-arid area with sandy loam soil. The HG was applied at two doses (0 and 45 kg ha^−1^) at the time of sowing, at a depth of 20 cm, while fulvic acid was applied foliarly at concentrations of 1 and 2 g L^−1^. The results showed that grain yield increased from 8.3 t ha^−1^ in the treatment without HG to 9.4 t ha^−1^ when this material was incorporated. This increase was mainly attributed to the improvement in the water retention capacity of the sandy loam soil, the reduction of losses by percolation, and the consequent decrease in water stress in plants.

The life cycles reported for HGs vary mainly according to the type of material used. In general, HGs of biological origin present a shorter lifespan due to their higher susceptibility to microbial degradation in soil, compared to those of synthetic origin, which tend to show greater structural stability. However, beyond the type of material, the duration and effectiveness of HGs also depend on environmental conditions, soil characteristics, and application time. Kumar et al. [95] estimated that the lifespan of HGs can range between 2 and 5 years, depending on edaphic and climatic factors. Likewise, Yang et al. [98] observed that the application of superabsorbent polymers during biennial cycles generated higher yields in the second year of evaluation, which was attributed to a cumulative effect of the material in the soil.

The life cycle of HGs is critical due to the increase in costs in crop systems. In the case of HGs of biological origin, their degradation is much faster compared to those produced with synthetic materials [99,100]. However, the costs of restoring soils degraded by intensive agriculture vary depending on the impact on the soil and the restoration strategy, with an approximate estimate of 185 USD/ha to 3012 USD/ha [101,102]. In addition, the restoration time of degraded soils is more than ≥10 years [103]. Therefore, adopting the use of HGs in cropping systems represents a functional alternative to mitigate degradation processes by improving water use efficiency, with positive effects on both system sustainability and crop productivity. Nevertheless, the long-term agronomic and environmental performance of hydrogels remains insufficiently documented under field conditions. In particular, further studies are required to evaluate their persistence in soil, biodegradation behavior, and cost-effectiveness under different cropping systems before their large-scale adoption can be fully supported.

## 4. Recent Research Trends in the Encapsulation of Microorganisms, Bioactive Particles, and Agrochemicals in Hydrogel Spheres

The integration of bioactive compounds into hydrogel matrices can be achieved through various encapsulation strategies, among which coacervation [20] and emulsification [104] are the most widely used because of their ability to protect functional agents and modulate their release from polymeric matrices. As a result, encapsulation in HG spheres has emerged as a promising strategy for the retention and controlled release of a wide range of functional agents, including microorganisms, bioactive particles, and agrochemical compounds.

In the case of microorganisms, this technique has been widely applied to improve their stability, viability, and gradual release in soil or in the rhizosphere [105]. Several studies have reported the encapsulation of species with agricultural potential, including *Trichoderma harzianum* [106], *Azospirillum brasilense Sp7 and FP2* (wild type) [42,107], *Acidithiobacillus thiooxidans* [41], and *Bacillus subtilis* [108]. Although each study presents specific objectives, the common purpose is to improve agricultural productivity, particularly the increase in various fruit, horticultural, and legume crops, among others.

A key indicator in functional HGs is encapsulation efficiency, as previously discussed. This is determined by the number of colony-forming units in agar medium and reflects the degree of effective immobilization of the microorganism [15]. However, the effectiveness of these systems does not depend solely on the physical encapsulation capacity but also on the physiological and metabolic characteristics of the strains used. In this regard, genetic engineering and gene editing tools have begun to be employed to optimize the performance of encapsulated microorganisms and enhance their beneficial functions in soil. For example, Shafi et al. [109] described advances in the molecular manipulation of plant growth-promoting bacteria (PGPR) using CRISPR-Cas systems, aimed at improving the production of siderophores, key compounds in iron capture and availability in the rhizosphere. The study by Gou et al. [110] constitutes another example of the potential of genetic engineering applied to PGPR. In this work, the authors genetically modified Bacillus strains to increase the production of antimicrobial compounds, which significantly enhanced their ability to inhibit phytopathogenic bacteria. Similarly, Nie et al. [111] demonstrated that tools based on CRISPR systems can be used to reprogram the metabolism of Pseudomonas chlororaphis, allowing optimization of the synthesis of metabolites associated with biocontrol and adaptation to environmental stress conditions. Another approach carried out by Chauhan et al. [112] employed *B. amyloliquefaciens* SN13 and rice (*Oryza sativa*) as a model system to unravel the regulatory networks governing plant–PGPR interaction under saline stress. Inoculation with SN13 increased biomass, relative water content, and accumulation of proline and soluble sugars, while reducing lipid peroxidation and electrolyte leakage, demonstrating a substantial physiological improvement under salinity through omics techniques. These strategies allow the design of microbial strains with greater functional capacity, which is particularly relevant when used in HG encapsulation systems, as it improves the biological efficiency of the inoculant and its performance after release in soil.

Genetic modifications in bacteria to improve their capacity for producing metabolites of interest are promising; however, the use of tools such as CRISPR-Cas9 is not limited only to microorganisms but has also been applied in plants, offering the possibility of increasing their resilience, productivity, and synthesis of bioactive compounds [113,114,115]. Nevertheless, their large-scale implementation raises important ethical and regulatory issues, such as possible unintended effects on ecosystems, gene transfer to wild species, impacts on biodiversity, and inequalities in access to technological benefits [116]. Due to these uncertainties and heterogeneous regulatory frameworks, some countries adopt a precautionary stance and restrict or do not authorize the cultivation of genetically edited plants using CRISPR-Cas9 [117].

The encapsulation of PGPR or other microorganisms in HG matrices offers an alternative strategy to protect modified or unmodified microorganisms and preserve their viability and release their compounds gradually in the rhizosphere. This technique optimizes their biostimulant and biocontrol activity in soils under abiotic stress, constituting an efficient tool to strengthen soil health and improve agricultural productivity.

On the other hand, HGs can encapsulate bioactive particles derived from microalgae, which include a wide variety of active compounds such as carotenoids, polysaccharides, bioactive peptides, and vitamins [118]. Likewise, other metabolites derived from microalgae with biological activity are biostimulants that not only regulate physiological processes but also optimize crop productivity, improve nutrient absorption, reduce fertilizer use, and increase their efficiency [119,120]. Microalgal biomass has a high content of phytohormones considered as biological biostimulants, rich in auxins, cytokinins, gibberellins, abscisic acid, ethylene, brassinosteroids, salicylic acid, jasmonic acid, and strigolactones [121]. The incorporation of these microalgae-based biostimulants for crop improvement is a recent field of study [122,123,124] and is considered an efficient ecological alternative to reduce soil degradation and strengthen crop resilience to abiotic stress [125]. However, it presents some limitations in use derived from the lack of standardization in formulations, generating inconsistent results [126] and the absence of regulation. This lack of regulatory frameworks limits their comparative evaluation and commercial acceptance [127].

Finally, the encapsulation of agrochemicals in HGs made from biodegradable materials constitutes a promising strategy for the controlled release of essential nutrients in agriculture, with nitrogen being one of the most relevant nutrients for plant growth. Among nitrogen sources, urea has been widely studied in encapsulation systems to improve its efficiency and reduce losses by leaching. In one approach, Pandya & Mungray et al. [128] applied ionic gelation in a chitosan matrix with a carrageenan core (CK-CU 10), achieving a swelling index of 121% and a controlled urea release of 70.4% from an initial concentration of 0.350 g. Similarly, other studies have employed alginate matrices for the slow release of urea, showing improvements in nutrient efficiency and its gradual availability for crops [129].

In addition, organic fertilizers have not only been encapsulated, but the construction of an intelligent pesticide release system such as B-cyhalothrin has also been reported, with characteristics sensitive to environmental stimulation in an HG matrix called OMt/Alg/PNIPAM with an 82.06% pesticide loading and a release of 3.9%, minimizing environmental contamination [130]. The pesticide dichlorvos was encapsulated in an HG matrix from a 2000 ppm pesticide solution. Its release was 1024.4 ppm after 44 h, with a non-Fickian diffusion mechanism, meaning that the release rate of dichlorvos was equivalent to the relaxation time of the HG matrix [131]. The possibilities for encapsulating different compounds are subject to the needs of farmers within the framework of sustainability and improvement of agricultural production. Table 2 summarizes the most important articles on the encapsulation of microorganisms, bioactive particles, and agrochemicals.

The comprehensive analysis of Table 2 demonstrates a paradigmatic evolution in the design of materials for sustainable agriculture, transitioning from purely water-retaining soil conditioners toward smart and multifunctional matrix systems. At the core of this technology lies the close relationship between the chemical composition of the polymeric matrix and its physicochemical properties. A well-defined contrast is observed between synthetic acrylic polymers modified with cellulosic nanofillers, such as interpenetrating poly (AAm co AA) networks with cellulose nanocrystals or nanofibers, and matrices composed of natural biopolymers such as alginate, chitosan, starch, or Aloe vera. While crosslinked synthetic matrices achieve massive swelling indices ranging from 14,700% to 41,200%, natural biopolymers exhibit more moderate water absorption capacities (between 121% and 2630%). Nevertheless, this lower water retention in natural matrices is offset by significantly superior biocompatibility, a fundamental aspect for the cellular immobilization of beneficial microorganisms.

Matrices formulated from modified polysaccharides display biodegradation rates between 40% and 94% over periods ranging from two weeks to three months, positioning them as ecologically safe alternatives. However, examining the application scale in the current literature reveals a critical gap for industrial scaling up: more than 80% of research is limited to laboratory trials or pot experiments under greenhouse conditions, with a scarce representation of open field studies.

## 5. Environmental Precautions of Hydrogels in Soils

Although numerous advantages and benefits of using HGs in agricultural applications have been described, concern regarding the environmental impact generated by their degradation byproducts must be carefully considered. To minimize this ecological footprint, numerous studies have focused on the sustainable design of HGs, prioritizing the biodegradability and bioinert nature of the materials [137]. Among these, hybrid polymers designed to release inert and nontoxic compounds upon degradation stand out, providing farmers with the certainty that seasonal HG application will not result in a gradual accumulation of polymeric residues in the soil.

Other strategies considered to mitigate these impacts involve applying green chemistry principles and integrating life cycle assessment (LCA) during the functional polymer design phase [138]. Likewise, special attention must be paid to the development and environmental behavior of functional HGs, as their interaction with the soil matrix can trigger adverse effects such as unwanted nutrient immobilization or alterations in edaphic microbial communities [139]. Evaluating these factors is essential to guarantee the holistic sustainability of this technology.

## 6. Critical Assessment, Contradictory Findings, and Research Gaps

Although hydrogels have demonstrated considerable potential for improving water retention and controlled release in agriculture, several inconsistencies remain regarding their large-scale implementation. One of the main controversies concerns their economic viability. Some studies have reported positive benefit–cost ratios associated with reduced irrigation requirements and increased crop productivity, particularly under water-limited conditions. For example, Songara and Patel [140] reported that guar gum-based hydrogels increased crop yield by 5.5–8.0%, improved soil moisture by 10.5–17.0%, and the benefit–cost ratios of the 2.5 and 5 kg ha−1 guar-gum-based hydrogels were 2.7 and 2.1, respectively. In contrast, other studies found that hydrogel application reduced the economic return compared with conventional practices because of the high initial cost of the material, particularly in developing countries [141,142,143]. For instance, the benefit–cost ratio declined from 2.15 in the untreated control to 1.86, 1.72, 1.52, 1.36, and 1.24 following hydrogel applications of 10, 15, 20, 25, and 30 kg ha^−1^, respectively, indicating that increasing application rates does not necessarily improve economic profitability [141].

Another important discrepancy concerns hydrogel performance under laboratory and field conditions. Water absorption capacities commonly reported under distilled water conditions often exceed 1400–600 g g^−1^, whereas values measured under agricultural soils are frequently below 100 g g^−1^ because of salinity, ionic strength, soil compaction, and multivalent cations [144,145]. These differences suggest that laboratory results cannot be directly extrapolated to field performance and highlight the need for standardized evaluation protocols under realistic agroenvironmental conditions.

Environmental sustainability also remains controversial. While biodegradable hydrogels have been proposed as environmentally friendly alternatives, their degradation rate varies considerably depending on soil properties, temperature, and microbial activity [146]. Conversely, synthetic hydrogels may persist in soil, generating residues or microplastic-like fragments and potentially altering soil microbial communities, aeration, and nutrient cycling [147].

## 7. Challenges in Hydrogel Development and Opportunities to Improve Agricultural Production and Combat Water and Abiotic Stress

To some extent, HG technology meets the basic requirements in sustainable agriculture (reducing the water footprint and enabling controlled release of certain substances) that must be considered within strategies to increase agricultural production and reduce mineral fertilizers. The type of material used to produce the HG is a key element in selecting certain components to be encapsulated, improving their efficiency and their intrinsic property related to the diffusion mechanism [74,148]. This review has analyzed the different types of particles that can be encapsulated in HGs. It is important to highlight that compounds of biological origin have greater environmental acceptance based on the development of the bioeconomy, driven by the demand for more sustainable products with high efficiency and performance in agricultural soils. Biodegradable HGs with bioactive particles should be implemented in agricultural systems. In an initial approach, Athanasiou et al. [149] synthesized an alginate-based HG encapsulating melatonin as a biostimulant agent, which was tested on *Solanum lycopersicum* seeds under saline stress (50 and 70 mM) and without saline stress. The results showed that plants were 24% and 16% longer under 50 and 70 mM NaCl, respectively. This latter approach demonstrates the feasibility of HGs to encapsulate nutrients and/or biostimulants to improve crop production.

As is well known, technological evolution and global challenges are constantly changing in the agricultural sector. The development of these intelligent systems for the controlled release of agrochemicals and beneficial microorganisms is a reality [150,]. However, some nano-encapsulation approaches are not suitable because their long-term effects on soil health and ecosystems have not been evaluated [151]. On the other hand, the encapsulation of microorganisms such as *Bacillus* sp. [152] and *Trichoderma* spp. [19] is beneficial, but their preparation and the determination of long-term storage conditions and shelf life are stages that are often omitted, focusing on freshly prepared structures or not providing such information. Therefore, more research is needed to understand the transfer mechanisms of some components in order to optimize plant regulatory growth.

Another critical point is the development and application of HGs in agriculture, as they face barriers that limit both industrial scaling and functional stability in agricultural soils, especially when synthetic matrices are used. Nevertheless, several field-scale studies have demonstrated promising agronomic performance under real agricultural conditions. Smagin et al. [153] reported increases of 30–50% in plant productivity and biomass quality, together with 1.3–2-fold water savings and protection against secondary soil salinization across contrasting climatic and soil conditions. Similarly, Azimov et al. [154] validated hydrogel application over 450 ha of irrigated, rainfed, and arid agricultural land, with a 7.8% increase in soil water-holding capacity, 17.2% higher seed germination under arid conditions, and a 21.6% increase in plant biomass under irrigation (95% CI: 17.9–25.3%). Likewise, under a randomized complete block field experiment conducted over two consecutive growing seasons, El-Aziz et al. [155] achieved the highest rice yield (10.76 t ha^−1^) and fresh clover forage yield (5.02 t ha^−1^) using a nanosilica hydrogel with 90% irrigation and 119 kg ha^−1^ hydrogel application, while simultaneously improving soil water retention and water productivity. Although these studies provide valuable field evidence, they remain limited to specific crops, climatic conditions, and hydrogel formulations. Despite these encouraging results, hydrogel performance under complex edaphic conditions remains insufficiently understood, particularly where water scarcity coexists with salinity or sodicity [148,156]. These challenges highlight the need not only for broader field validation but also for a better understanding of the physiological mechanisms underlying crop responses to hydrogel application.

At this point, it should be noted that plant tolerance and resilience depend on finely regulated physiological mechanisms, particularly phytohormonal homeostasis, whose alteration can accelerate senescence processes and cause irreversible wilting. The modulation of these regulatory pathways is therefore a determining factor in mitigating damage induced by adverse environmental conditions. Phytohormones govern plant growth and development under normal conditions and play a central and irreplaceable role in signal transduction pathways activated during environmental stress [157,158]. In addition, the challenges of modern agriculture, such as water scarcity, soil degradation, and climate change, reinforce the need to enhance these materials.

The future approach is that HGs should be responsive to multiple stimuli and capable of adapting simultaneously to changes in temperature, pH, and ionic strength in order to improve plant adaptability and resistance to abiotic and water stress. In an initial approach, Chandrika et al. [159] prepared a pH-sensitive superabsorbent HG based on crosslinked guar gum-g-polyacrylate. These HGs contain hydrophilic groups (-COOH and -COO^−^), which are more ionized at pH 9.0, generating electrostatic repulsion that leads to the expansion of the polymer network. This latter approach has demonstrated the feasibility of HGs in acidic or alkaline soils for nutrient retention and optimal performance in such soils. More recent studies have expanded these initial advances; for example, Wu et al. synthesized a novel biodegradable semi-interpenetrating polymer network HG sensitive to pH, designed for arid environments with a water retention of 272.01 g/g. The release of nitrogen, phosphorus, and potassium was pH-dependent, with slower release under acidic conditions (pH 4), attributed to protonation/deprotonation, fitting the Korsmeyer–Peppas model.

Overall, these advances demonstrate the potential of multifunctional HGs to promote more efficient and sustainable resource use in agriculture, particularly in developing regions where soil degradation constitutes a critical limitation. This scenario is especially relevant in coastal areas, such as in the case of Peru, where agricultural activity has increased in recent years [9]. However, significant challenges remain, such as the increase in soil salinization associated with the intensive use of synthetic fertilizers and the reuse of wastewater for irrigation [160], which affects the efficiency and stability of these materials under real conditions. Therefore, to apply hydrogels to improve crop performance, researchers should initiate testing not only with a single evaluation factor but also under real conditions or a combination of both climatic and edaphic factors, since a decrease in swelling capacity has been observed when there is a high concentration of ions, limiting both water retention and the controlled release of bioactive compounds [149,161]. Likewise, extreme pH conditions affect the ionization of hydrogel functional groups, modifying their structure and release kinetics [31]. Among the different types of hydrogels, those based on polysaccharides such as cellulose and chitosan stand out for their biodegradability and affinity for bioactive compounds [19,162], and can operate through sorption–diffusion mechanisms. However, their high susceptibility to degradation and limited mechanical stability under adverse environmental conditions remain relevant limitations. Therefore, future research should focus on the development of hybrid or composite materials that balance biodegradability, stability, and functionality.

## 8. Conclusions and Future Perspectives

This review conducted a comprehensive analysis of the development and application of HGs in agriculture, addressing their scientific evolution, technological maturity level, functionality as delivery systems, and their role in the encapsulation of bioactive compounds, microorganisms, and agrochemicals. Based on bibliometric and technical analyses, clear trends were identified that demonstrate the consolidation of this technology as an emerging tool to address challenges associated with water stress and soil degradation. The following conclusions are derived from the critical analysis of current advances in the intensification of agricultural systems based on HGs.

1. Sustained growth in publications, especially since 2020, reflects increasing interest in the use of HGs as superabsorbent materials and controlled-release systems. However, most developments are at intermediate levels of technological maturity (TRL 3–4), highlighting a significant gap between laboratory-scale research and commercial-scale implementation. In this context, scalability, operational stability, and economic viability remain priority challenges.
2. The performance of HGs depends on multiple factors, among which formulation parameters are the most important. These determine the efficiency of the material under real field conditions to improve water use and reduce losses due to evaporation and percolation, which is especially relevant in sandy soils or under low precipitation conditions.
3. The encapsulation of PGPR, compounds derived from microalgae, and agrochemicals represents a rapidly expanding line of research. These strategies are promising because they improve the stability, viability, and effectiveness of bioactive agents in the soil. In particular, the encapsulation of microalgae-derived biostimulants and phytohormones emerges as an ecological alternative to modulate key physiological pathways in plants subjected to abiotic stress. However, the lack of standardization in formulations and the absence of clear regulatory protocols limit their scalability and commercial acceptance.
4. Despite their agricultural advantages, significant technical, economic, and environmental limitations persist. In materials science, a major trade-off exists between biodegradability and functional durability, which limits their lifespan and leads to variable performance depending on soil type. Likewise, long-term field data regarding their impact on soil health and nutrient dynamics are required, along with standardized regulatory frameworks that ensure the safety of novel formulations. Looking forward, overcoming these barriers will require a multidisciplinary approach. The field will benefit from integrating multiomic approaches (metagenomics and metabolomics) to evaluate rhizosphere interactions, combined with life cycle assessment (LCA) and technoeconomic studies. Finally, scaling up from the experimental phase to the commercial market will require establishing demonstration networks in real agricultural fields alongside government incentives, such as subsidies or carbon credits.

In conclusion, agricultural hydrogels represent a strategic technological platform to advance toward more efficient and sustainable agricultural production. Although advances in their chemical design and functionality are highly promising, their ultimate integration into the agricultural sector will depend on achieving an optimal balance between water and nutrient management efficiency, economic viability for farmers, and long-term ecological safety.

## Figures and Tables

**Table 1:** Summary of development approaches for inventions in the field of “hydrogel synthesis” AND “agriculture ^1^”.

Development Approach	Patent ^1^
Synthesis of HGs for agriculture and other applications	WO2025091138A1, RO134967A2, CN104530336A, US2010234233A1, AU2013204055A1, CN106750379A, CN101857655A, CN113603528A, US2015376300A1, CN101555306A, JP2007070473A, RS20190959A2, JP2009270048A, CN118420930A, KR102555843B1, CN115716924A US12528766B2, AU2016219713A1
Encapsulation of nutrients and other components	RO132657A2, US2020283601A1, US2020262765A1, KR100671965B1, CN110478525A, RO128903A0, US2024196887A1, US2014335184A1
Additives or design improvements	CN106750392A, CN106750391A, CN106750378A, CN106349487A, JP2009046553A, JP2008074924A, JP2010018665A, CN119241767A, US12269785B2, KR20210018828A, JP2008074925A, JP2002053629A, JP2002053762A, DE10037293A1
Synthesis of HGs as adsorbents of metals and other contaminants	CN120022819A, CN106311188A, WO9940990A1, KR100423575B1
Other patents	US2024336842A1, JP2026503870A, US20260028612A1, US20260021142A1, US2025303006A1, US2025011193A1, CN115572202A, US2022033804A1, US2023113447A1, US12201643B2, US2022161233A1, KR20210034544A, AU2014271268A1, US2018289043A1,

^1^ Source: Espacenet, using “hydrogel synthesis” AND “agriculture” as a keyword; search conducted on 31 May 2025.

**Table 2:** Encapsulation of microorganisms, bioactive particles and agrochemicals in hydrogel matrices.

N°	Polymer Matrix	Encapsulated Bioactive Compound	Classification	Swelling Index (%)	Deswelling Studies (%)	Release Rate (%)	Biodegradation Rate	Crop Type	Performance Indicators	Soil Type	Application Scale	Reference
1	Chitosan (2% *v*/*v*)	Urea (10% dosage)	Releaser	121	21.22	70.4	2–3 weeks	Jasmine pot	NP	NP	Laboratory	
2	Sodium polyacrylate	Nitrogenous fertilizers (N) (NH_4_^+^, NO_3_^−^, and urea)	Releaser	NP	NP	NP	NP	Bok Choy (*Brassica rapa* var. *chinensis*)	Plant height (54%) Fresh weight (FW) (119.65%) Dry weight (DW) (170.16%)	Sand (94.18%) Clay (3.32%) Silt (2.5%)	Greenhouse pot	
3	Sodium alginate (2% *v*/*v*)	*Rhizophagus irregularis*, *Rhizophagus intraradices*, and *Funneliformis mosseae*	Encapsulant	NP	NP	NP	NP	NP	Germination rate (64.3%)	NP	Laboratory	
4	Poly(acrylamide-co-acrylic acid) Sodium alginate Cellulose nanocrystals	Urea (commercial)	Releaser	41,200	NP	86	NP	Tomato	Leaf chlorophyll (16.74%) Leaf number (8.21%) Stem diameter (6.84%) Plant height (8.83%)	Sand (79.5%) Clay (13.2%) Silt (7.3%)	Greenhouse pot	
5	Sodium alginate Poly(N-isopropylacrylamide) Organomontmorillonite (OMt)	λ-cyhalothrin (LC/λ-cyhalothrin)	Adsorbent-releaser	1100	NP	3.9	12 weeks	NP	NP	NP	NP	
6	Carboxymethylcellulose (CMC) Cellulose Nanofibers (CNF)	Urea	Adsorbent-releaser	14,700	18	90 (20–30 days)	NP	Wheatgrass plants	Stem length (80.7%) Root length (97.4%) Fresh weight (FW: 75.2%) Dry weight (DW: 63%)	Sand Silt	Greenhouse pot	[132]
7	Aloe vera Acrylic acid (AA)	Dichlorvos	Adsorbent-releaser	756	NP	51.22% (44 h)	94% (10 weeks)	NP	NP	NP	Laboratory	
8	Modified Colocasia esculenta starch Polyacrylamide (PAAm)	Urea Ammonium sulfate Potassium nitrate Biofertilizer (fortified nutrient)	Adsorbent-releaser	1356	NP	76.5% urea 69.7% ammonium sulfate 73.2% potassium nitrate 60% biofertilizer (fortified nutrient)	76% (70 days)	NP	NP	NP	Laboratory	[133]
9	Chitosan (CS) PE@CS Carboxymethylchitosan (CMCS) CMCS-NP	Penconazole (PE)	Releaser	NP	NP	5.27% PE@CS 89.13% CMCS-NP	NP	*C. plumeria* inhibition	20% inhibition	NP	Laboratory	[134]
10	Superabsorbent polymer	*Trichoderma*	Encapsulant	NP	NP	NP	NP	Rainfed rice varieties	Number of productive tillers (24.15%) Number of grains per panicle (8.26%) Dry weight (DW: 6.45%)	NP	Field/Cropland	
11	Sodium alginate (SA) alone Xylan (Xyl)	Zinc Oxide Nanoparticles (ZnONPs) *Azospirillum brasilense*	Adsorbent-releaser	NP	NP	1.7% zinc 2.1% zinc + A. brasilense	46% (60 days)	Maize plants	Plant height (110.5%) Number of leaves (48%) Stem diameter (13.2%) Root (116%) Dry weight (DW: 24%)	NP	Greenhouse pot	[135]
12	Chitosan (Cht) Alginate (Alg) Cenosphaeres (Cn)	Imidacloprid (IMI)	Adsorbent-releaser	2630	NP	80% (72 h)	40%	*Vigna unguiculata* *Vigna radiata*	Germination (66%) Shoot length (81.2%) Root length (82.4%) Pest control (100%)	NP	Greenhouse pot	[136]

NP: Not reported.

## Data Availability

The articles downloaded from Scopus and the results of the VOSviewer analyses are provided as Supplementary Materials. No new experimental data was generated.

## Supplementary Materials

The following supporting information can be downloaded at: https://www.mdpi.com/article/10.3390/gels12090785/s1, Table S1: Scopus records retrieved from the systematic literature search (743 records), articles selected for VOSviewer analysis (548 records), Espacenet search query, and patent records identified in Espacenet (58 patents).
